# Enantioselective Synthesis of the Ethyl Analog of the Marine Alkaloid Haliclorensin C

**DOI:** 10.3390/molecules24061069

**Published:** 2019-03-18

**Authors:** Guillaume Guignard, Núria Llor, David Pubill, Joan Bosch, Mercedes Amat

**Affiliations:** 1 Laboratory of Organic Chemistry, Department of Pharmacology, Toxicology, and Therapeutic Chemistry, Faculty of Pharmacy and Food Sciences, and Institute of Biomedicine (IBUB), University of Barcelona, 08028 Barcelona, Spain; guignard.guillaume@gmail.com (G.G.); nllor@ub.edu (N.L.); joanbosch@ub.edu (J.B.); 2 Pharmacology Section, Department of Pharmacology, Toxicology, and Therapeutic Chemistry, Faculty of Pharmacy and Food Sciences, and Institute of Biomedicine (IBUB), University of Barcelona, 08028 Barcelona, Spain; d.pubill@ub.edu

**Keywords:** alkaloids, macrocycle, amino alcohols, ring opening, reduction, enantioselective synthesis

## Abstract

The enantioselective synthesis (3.7% overall yield in nine steps from **2**) and biological screening of the ethyl analog of the macrocyclic marine alkaloid haliclorensin C (compound **5**) are reported. Amino alcohol **3**, generated by a LiNH_2_BH_3_-promoted reductive ring-opening/debenzylation sequence from phenylglycinol-derived lactam **2**, was used as the starting chiral linear building block. Incorporation of the undecene chain via the nosyl derivative **12**, methylenation of the pentanol moiety, and a ring-closing metathesis are the key steps of the synthesis.

## 1. Introduction

**Figure 1:**
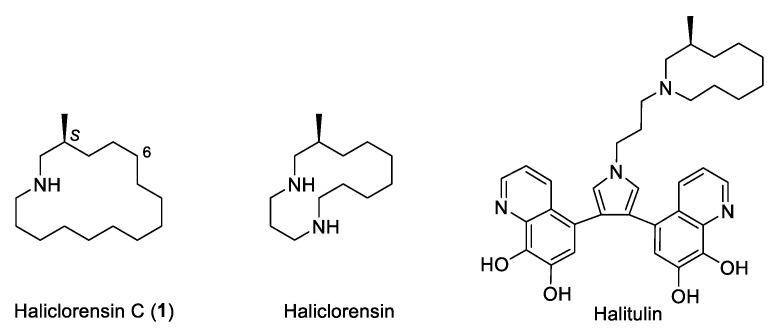
Azamacrocyclic marine alkaloids isolated from *Haliclona tulearensis*.

Haliclorensin C (**1**) is a minor marine alkaloid isolated from the sponge *Haliclona tulearensis* collected in January 2006 in Salary Bay, Madagascar [1]. From 86 g of a frozen wet sample of sponge, only 2 mg (0.002 wt.%) of haliclorensin C (**1**) was isolated. The structure of **1** was assigned as 3-methylazacyclohexadecane from its ^1^H- and ^13^C-NMR spectroscopic data, with the aid of HMBC and COSY experiments, and mass spectrometry data (HREIMS). Based on biogenetic considerations, the stereogenic center of haliclorensin C was tentatively proposed to possess the *S* absolute configuration as previously determined for haliclorensin and halitulin, two related azamacrocyclic alkaloids isolated from the same marine sponge [2,3]. Haliclorensin C exhibited moderate toxicity in the brine shrimp (*Artemia salina*) test (LD_50_ value 2.1 mM) [1], whereas haliclorensin and halitulin were shown to have significant cytotoxicity, the former against P-388 mouse leukemia cells [4] and the latter against several tumor cell lines [5] (Figure 1).

The proposed structure for haliclorensin C was confirmed in 2014 by total synthesis [6], although comparison of the ^1^H- and ^13^C-NMR spectra of both the base and hydrochloride of the synthetic product with the spectra reported for the natural product revealed that the isolated sample of haliclorensin C corresponded to a protonated sample. From the stereochemical standpoint, the synthesis relies on the use of a chiral amino alcohol building block (fragment N1-C6 of haliclorensin C) generated from a phenylglycinol-derived oxazolopiperidone lactam that contains the required *S* stereocenter at the β carbon to the amino group.

In recent years we have reported the preparation of enantiopure five-carbon linear building blocks from phenylglycinol-derived bicyclic lactam scaffolds and their application to the enantioselective total synthesis of macrocyclic natural products, such as haliclorensin marine alkaloids [6], fluvirucinin B_1_ [7], and callyspongiolide [8]. Our approach involves the LiNH_2_BH_3_-promoted reductive opening of the oxazolidine and lactam rings of the starting oxazolopiperidone lactam, and the subsequent reductive [9] or oxidative [7] removal of the chiral inductor to give diversely substituted 5-aminopentanols [9], 5-hydroxypentanoic acids or 5-hydroxypentanenitriles [7]. Taking into account the availability of starting lactams with a variety of substitution and stereochemical patterns [10,11], the above methodology provides easy access to a range of useful functionalized chiral linear building blocks with potential application in the enantioselective synthesis of natural product analogs.

## 2. Results and Discussion

We herein present the enantioselective synthesis of the ethyl analog of haliclorensin C (compound **5**) and the results of its in vitro screening in a panel of biological assays. Marine natural products and their modified derivatives have long been recognized as one of the most important sources of new biologically active substances and therapeutic agents [12,13].

**Scheme 1:**
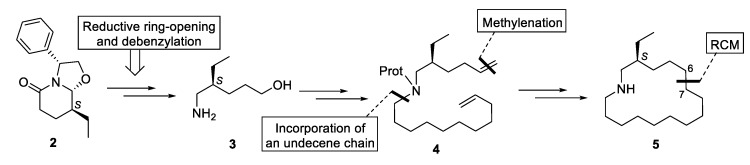
Synthetic strategy.

The assembly of the azacyclohexadecane ring of **5** would be accomplished by a ring-closing metathesis (RCM) reaction of an appropriate *N*-(hexenyl)undecenylamine **4** (bond formed C6-C7), which would be prepared from amino alcohol **3**, easily available from bicyclic lactam **2**, by incorporation of a C_11_ chain bearing a terminal double bond and subsequent methylenation of the pentanol moiety (Scheme 1).

**Scheme 2:**
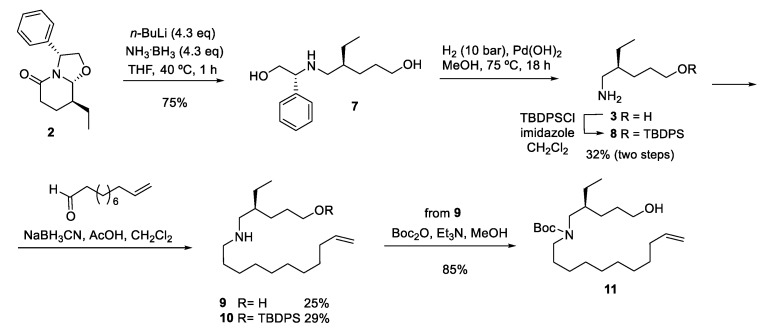
First approach to the ethyl analog of haliclorensin C.

Lactam **2**, bearing the required ethyl substituent with the appropriate stereochemistry, was efficiently converted (75% yield) to amino diol **7** [9] by treatment with lithium amidotrihydroborate (LiNH_2_BH_3_) [14,15] generated in situ by deprotonation of the commercially available borane-ammonia complex with *n*-BuLi. This transformation involves an initial hydride attack on the lactam carbonyl, followed by a Grob-type fragmentation resulting in a double ring-opening by cleavage of the C–N and C–O bonds, with a final hydride reduction of the resulting imino aldehyde intermediate. Unfortunately, removal of the chiral inductor of **7** by hydrogenolysis, followed by silylation of the resulting crude amino alcohol **3**, afforded the primary amine **8** in low overall yield (Scheme 2). In a first approach to compound **5**, the undecene chain was introduced by reductive amination with 10-undecenal. Starting from either the crude amino alcohol **3** or the *O*-protected derivative **8**, the corresponding secondary amines **9** or **10** were obtained, but also in low yield. For this reason, although the amino group of **9** was satisfactorily protected as the *N*-Boc derivative **11**, this route was abandoned.

**Scheme 3:**
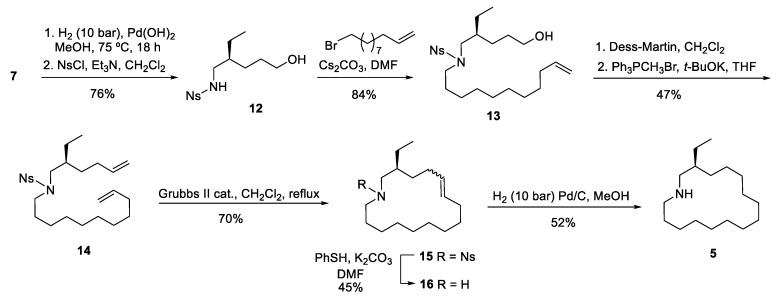
Synthesis of the ethyl analog of haliclorensin C (**5**).

To overcome the above drawbacks, we then focused on an alternative approach in which the undecene chain would be introduced by alkylation of the known [6] nosyl-protected amine **12** with undecenyl bromide. This activated amine was satisfactorily prepared in 76% overall yield by debenzylation of amino diol **7** by hydrogenolysis, followed by reaction of the resulting crude amino alcohol **3** with *o*-nitrobenzenesulfonyl chloride (Scheme 3). The alkylation step was performed in the presence of Cs_2_CO_3_, leading to alcohol **13** in excellent yield (84%). A subsequent Dess-Martin oxidation, followed by a Wittig methylenation of the resulting aldehyde, afforded *N*-(hexenyl)undecenylamine **14** in 47% overall yield. Macrocyclization of diene **14** took place satisfactorily by ring-closing metathesis using the second-generation Grubbs catalyst in refluxing dichloromethane under dilute solvent conditions, providing a 88:12 diastereoisomeric mixture of *E*/*Z* cycloalkenes **15** in 70% yield. Removal of the nosyl group by treatment with thiophenol, followed by catalytic hydrogenation of the resulting mixture of azamacrocyclic alkenes **16**, afforded the ethyl analog of haloclorensin C (**5**).

Compound **5** was submitted to biological screening in the context of the Lilly Open Innovation Drug Discovery (OIDD) program, where it was subjected to a battery of assays against new potential therapeutic targets. Among all the tests performed, the most pharmacologically relevant results were obtained in the oncology area. Specifically, compound **5** caused 30% inhibition of SETD8 at a concentration of 10 μM, measured through a scintillation proximity assay (SPA) of enzyme inhibition [16]. SETD8 is a lysine methyltranferase that methylates histone H4 at Lys 20. Its overactivation or overexpression has been found to play a role in the progression of certain cancers such as neuroblastoma [17]. Accordingly, inhibition of SETD8 in neuroblastoma leads to increased p53 tumor suppressor activity and reduced tumor cell growth, resulting in prolonged survival in mouse models of this neoplasia.

On the other hand, compound **5** yielded a 10.2% inhibition of cyclin-dependent kinase 2 (CDK2) at a concentration of 20 μM, measured through the SPA assay [18]. CDK2 is involved in cell cycle progression, and thus has been indirectly linked to cancer through its association with cyclin E, which activates it. Cyclin E binds to CDK2 to further phosphorylate the retinoblastoma proteins, which repress the E2F transcription factors, thus releasing and fully activating the E2Fs. E2Fs then trigger the transcription of S-phase proteins, including other cyclins, and promote cell cycle progression [19]. Cyclin E is frequently amplified in human tumors and is thought to promote proliferation and genome instability in several cancers.

In conclusion, the results herein reported further demonstrate the usefulness and versatility of chiral linear building blocks generated from phenylglycinol-derived oxazolopiperidone lactams in the synthesis of bioactive natural products and analogs. Our synthesis also illustrates the potential of ring-closing metathesis reactions for the efficient construction of azamacrocyclic rings [20,21,22]. In the light of the reported structure-activity relationships and inhibitory data of some SETD8 inhibitors [23], modifications of compound **5** could lead to a new series of SETD8 inhibitors with therapeutic potential. Finally, although the low inhibition of CDK2 caused by compound **5** rules out a potential therapeutic use based on this target, the possibility of a synergistic dual effect of this compound and its derivatives on SETD8 and CDK2 remains to be investigated.

## 3. Materials and Methods

### 3.1. General Information

All air sensitive manipulations were carried out under a dry argon or nitrogen atmosphere. THF and CH_2_Cl_2_ were dried using a column solvent purification system. Analytical thin-layer chromatography was performed on SiO_2_ (silica gel 60A 35–70 μm, Carlo Erba, Val de Reuil Cedex, France), and the spots were located with 1% aqueous KMnO_4_. Chromatography refers to flash chromatography, and was carried out on SiO_2_ (SDS silica gel 60 ACC, 35–75 mm, 230–240 mesh ASTM). Hydrogenations were carried out in a Parr 4560 high-pressure reactor. NMR spectra were recorded at 300 or 400 MHz (^1^H) and 100.6 MHz (^13^C), and chemical shifts are reported in δ values downfield from TMS, or relative to residual chloroform (7.26 ppm, 77.0 ppm) as an internal standard (see Supplementary Materials). Data are reported in the following manner: chemical shift, multiplicity, coupling constant (*J*) in hertz (Hz), integrated intensity, and assignment (when possible). Assignments are given only when they are derived from definitive two-dimensional NMR experiments (*g*-HSQC-COSY). IR spectra were performed in an Avatar 320 FT-IR spectrophotometer (Thermo Nicolet, Madison, WI, USA) and only noteworthy IR absorptions (cm^−1^) are listed. High resolution mass spectra (HMRS; LC/MSD TOF, Agilent Technologies, Santa Clara, CA, USA) were performed by Centres Científics i Tecnològics de la Universitat de Barcelona.

### 3.2. Experimental Procedures

*(S)-4-Ethyl-5-{[(1R)-2-hydroxy-1-phenylethyl]amino}-1-pentanol* (**7**). *n*-BuLi (3.90 mL of a 2.5 M solution in hexanes, 9.67 mmol) was added to a solution of the borane–ammonia complex (300 mg, 9.67 mmol) in anhydrous THF (6 mL) at 0 °C, and the resulting mixture was stirred at this temperature for 10 min and at room temperature for 15 min. This solution was added to a solution of lactam **2** [24] (550 mg, 2.25 mmol) in anhydrous THF (5 mL), and the stirring was continued at 40 °C for 1 h. The reaction mixture was quenched with H_2_O, and the resulting solution was extracted with Et_2_O. The combined organic extracts were dried over anhydrous Na_2_SO_4_ and filtered, and the solvent was removed under reduced pressure. Flash chromatography (from 8:2 hexane-EtOAc to 8:2 EtOAc-EtOH) of the residue gave amino diol **7** (425 mg, 75% yield) as a colorless oil: [α]^22^_D_ −44.9 (*c* 0.16, MeOH); IR (ATR Pike) ν (cm^−1^): 3330 (NH); ^1^H-NMR (400 MHz, CDCl_3_, COSY, *g*-HSQC): δ = 0.82 (t, *J* = 7.6 Hz, 3H, CH_3_), 1.23–1.40 (m, 3H, H-3, CH_3_C*H*_2_), 1.42–1.50 (m, 4H, H-2, H-3, H-4), 2.40 (dd, *J* = 11.6, 6.4 Hz, 1H, H-5), 2.46 (dd, *J* = 11.6, 5.0 Hz, 1H, H-5), 3.41 (br.s, 3H, OH, NH), 3.57–3.64 (m, 3H, H-1, CH_2_O), 3.71 (dd, *J* = 10.8, 4.0 Hz, 1H, CH_2_O), 3.77 (dd, *J* = 8.8, 4.0 Hz, 1H, CHN), 7.24–7.38 (m, 5H, ArH); ^13^C-NMR (100.6 MHz, CDCl_3_): δ = 11.3 (CH_3_), 24.9 (CH_3_*C*H_2_), 27.4 (C-3), 29.2 (C-2), 38.8 (C-4), 50.2 (C-5), 60.3 (C-1), 64.8 (CHN), 66.5 (CH_2_O), 127.3 (CH-Ar), 127.6 (CH-Ar), 128.6 (CH-Ar), 139.9 (C-*i*); HRMS (ESI) calcd for [C_15_H_25_NO_2_ + H^+^]: 252.1958, found. 252.1947.

(*S)-5-[(tert-Butyldiphenylsilyl)oxy]-2-ethyl-1-pentanamine* (**8**). A solution of aminodiol **7** (241 mg, 0.96 mmol) in anhydrous MeOH (10 mL) containing 20% Pd(OH)_2_ (48 mg) was hydrogenated at 75 °C for 18 h under 10 bar of pressure. The catalyst was removed by filtration and washed with hot MeOH. The organic solution was concentrated, and the resulting crude amino alcohol **3** was dissolved in CH_2_Cl_2_ (3 mL). Imidazole (271 mg, 3.98 mmol) and TBDPSCl (1.09 g, 3.98 mmol) were added, and the mixture was stirred at room temperature for 12 h and at 40 °C for 4 h. Saturated aqueous NH_4_Cl was added, and the mixture was extracted with CH_2_Cl_2_. The combined organic extracts were dried over anhydrous MgSO_4_ and filtered, and the solvent was removed under reduced pressure. Flash chromatography (from 95:5 hexane-EtOAc to EtOAc) of the residue gave amine **8** (115 mg, 32% yield) as a yellow oil: [α]^22^_D_ −5.78 (*c* 0.32, CHCl_3_); ^1^H-NMR (400 MHz, CDCl_3_, COSY, *g*-HSQC): δ = 0.88 (t, *J* = 7.4 Hz, 3H, C*H*_3_CH_2_), 1.04 [s, 9H, (CH_3_)_3_], 1.32–1.44 (m, 2H, H-3, CH_3_C*H*_2_), 1.45–1.58 (m, 4H, H-3, H-4, CH_3_C*H*_2_), 1.68–1.77 (m, 1H, H-2), 2.79 (dd, *J* = 12.8, 7.8 Hz, 1H, H-1), 2.89 (dd, *J* = 12.8, 5.4 Hz, 1H, H-1), 3.64 (t, *J* = 7.4 Hz, 2H, H-5), 7.34–7.43 (m, 6H, ArH), 7.63–7.66 (m, 2H, ArH), 7.70–7.73 (m, 2H, H_AR_); ^13^C-NMR (100.6 MHz, CDCl_3_): δ = 10.2 (*C*H_3_CH_2_), 19.3 [*C*(CH_3_)_3_], 23.6 (CH_2_), 26.7 (CH_2_), 27.0 [C(*C*H_3_)_3_], 29.2 (C-4), 37.5 (C-2), 42.7 (C-1), 63.9 (C-5), 127.8 (CH-Ar), 127.8 (CH-Ar), 129.7 (CH-Ar), 129.7 (CH-Ar), 134.0 (C-Ar), 134.9 (CH-Ar), 135.4 (C-Ar), 135.7 (CH-Ar).

*(S)-4-Ethyl-5-(10-undecenylamino)-1-pentanol* (**9**). A solution of amino diol **7** (370 mg, 1.47 mmol) in anhydrous MeOH (10 mL) containing 20% Pd(OH)_2_ (74 mg) was hydrogenated at 75 °C for 18 h under 10 bar of pressure. The catalyst was removed by filtration and washed with hot MeOH. The organic solution was concentrated, and the resulting crude amino alcohol **3** was dissolved in CH_2_Cl_2_ (1.3 mL). 10-Undecenal (0.58 mL, 2.94 mmol), NaBH_3_CN (277 mg, 4.42 mmol), and AcOH (0.13 mL) were added, and the mixture was stirred at room temperature for 12 h. Then, a saturated solution of NaHCO_3_ was added, and the mixture was extracted with CH_2_Cl_2_. The combined organic extracts were dried over anhydrous Na_2_SO_4_ and filtered, and the solvent was removed under reduced pressure. The residue was purified by flash chromatography (from 8:2 hexane-EtOAc to 8:2 EtOAc-MeOH) to afford secondary amine **9** (104 mg, 25% yield) as a colorless oil: [α]^22^_D_ −2.15 (*c* 0.3, MeOH); IR (ATR Pike) ν (cm^−1^): 2926 (NH); ^1^H-NMR (400 MHz, CDCl_3_, COSY, *g*-HSQC): δ = 0.89 (t, *J* = 7.4 Hz, 3H, C*H*_3_CH_2_), 1.21–1.69 (m, 21H, H-4, 10CH_2_), 1.99–2.05 (m, 2H, CH_2_=CHC*H*_2_), 2.56–2.61 (m, 1H, H-5), 2.66–2.75 (m, 3H, H-5, CH_2_N), 3.62 (m, 2H, H-1), 4.43 (br.s, 1H, NH), 4.89–5.00 (m, 2H, C*H*_2_=CH), 5.79 (dddd, *J* = 16.9, 10.2, 10.2, 6.7 Hz, 1H, CH_2_=C*H*); ^13^C-NMR (100.6 MHz, CDCl_3_): δ = 11.0 (*C*H_3_CH_2_), 24.9 (CH_2_), 27.1 (CH_2_), 27.1 (CH_2_), 28.3 (CH_2_), 28.6 (CH_2_), 28.9 (CH_2_), 29.1 (CH_2_), 29.3 (CH_2_), 29.3 (CH_2_), 29.5 (CH_2_), 33.8 (CH_2_=CH*C*H_2_), 37.7 (C-4), 49.5 (CH_2_N), 52.2 (C-5), 62.1 (C-1), 114.1 (*C*H_2_=CH), 139.1 (CH_2_=*C*H); HRMS (ESI) calcd for [C_18_H_37_NO + H^+^]: 284.2948, found: 284.2939.

*(S)-5-[(tert-Butyldiphenylsilyl)oxy]-2-ethyl-N-(10-undecenyl)-1-pentanamine* (**10**). 10-Undecenal (0.05 mL, 0.25 mmol) and AcOH (0.02 mL) were added to a suspension of amine **8** (74 mg, 0.20 mmol) and NaBH_3_CN (25 mg, 0.40 mmol) in anhydrous CH_2_Cl_2_ (2.3 mL), and the mixture was stirred at room temperature for 12 h. Then, water was added, and the mixture was extracted with CH_2_Cl_2_. The combined organic extracts were dried over anhydrous Na_2_SO_4_ and filtered, and the solvent was removed under reduced pressure. Flash chromatography (from 98:2 hexane-EtOAc to EtOAc) of the residue gave secondary amine **10** (30 mg, 29% yield) as a colorless oil: [α]^22^_D_ −12.3 (*c* 0.225, MeOH); ^1^H-NMR (400 MHz, CDCl_3_, COSY, *g*-HSQC): δ = 0.90 (t, *J* = 7.4 Hz, 3H, C*H*_3_CH_2_), 1.05 [s, 9H, (CH_3_)_3_], 1.24–1.58 (m, 18H, 9CH_2_), 1.65–1.77 (m, 3H, H-2, H-4), 2.01–2.06 (m, 2H, CH_2_=CHC*H*_2_), 2.81–2.84 (m, 2H, H-5), 2.88–2.96 (m, 2H, CH_2_N), 3.66–3.70 (m, 2H, H-1), 4.91–5.01 (m, 2H, C*H*_2_=CH), 5.80 (m, 1H, CH_2_=C*H*), 7.32–7.55 (m, 6H, ArH), 7.63–7.67 (m, 4H, ArH); ^13^C-NMR (100.6 MHz, CDCl_3_): δ = 10.2 (*C*H_3_CH_2_), 19.3 [*C*(CH_3_)_3_], 23.1 (CH_2_), 23.7 (CH_2_), 25.8 (CH_2_), 26.7 (CH_2_), 26.8 (CH_2_), 27.0 [C(*C*H_3_)_3_], 29.0 (CH_2_), 29.1 (CH_2_), 29.2 (CH_2_), 29.4 (CH_2_), 29.8 (CH_2_), 33.9 (CH_2_=CH*C*H_2_), 36.3 (C-4), 49.0 (CH_2_N), 51.6 (C-5), 63.8 (C-1), 114.3 (*C*H_2_=CH), 127.8 (CH-Ar), 129.8 (CH-Ar), 133.9 (C-Ar), 135.6 (CH-Ar), 139.3 (CH_2_=*C*H).

*(S)-4-Ethyl-5-[N-(tert-butoxycarbonyl)-N-(10-undecenyl)amino]-1-pentanol* (**11**). Et_3_N (0.04 mL, 0.28 mmol) was added to a solution of **9** (82 mg, 0.29 mmol) and di-*tert*-butyl dicarbonate (69 mg, 0.32 mmol) in anhydrous MeOH (1.5 mL), and the resulting solution was stirred for 12 h. Water was added, and the mixture was extracted with CH_2_Cl_2_. The combined organic extracts were dried over anhydrous MgSO_4_, filtered, and concentrated under reduced pressure. Flash chromatography (from 1:1 hexane-EtOAc to EtOAc) of the residue gave the *N*-Boc derivative **11** (95 mg, 85% yield) as a colorless oil: [α]^22^_D_ –0.82 (*c* 0.6, MeOH); IR (ATR Pike) ν (cm^−1^): 3453 (OH); 1694 (CO); ^1^H-NMR (400 MHz, CDCl_3_, COSY, *g*-HSQC): δ = 0.87 (t, *J* = 7.4 Hz, 3H, C*H*_3_CH_2_), 1.22–1.64 (m, 30H, C(CH_3_)_3_, H-4, 10CH_2_), 2.00–2.05 (m, 2H, CH_2_=CHC*H*_2_), 3.00–3.07 (m, 1H, H-5), 3.08–3.18 (m, 3H, H-5, CH_2_N), 3.54–3.64 (m, 2H, H-1), 4.89–5.00 (m, 2H, C*H*_2_=CH), 5.79 (dddd, *J*= 16.9, 10.2, 10.2, 6.7 Hz, 1H, CH_2_=C*H*); ^13^C-NMR (100.6 MHz, CDCl_3_): δ = 10.8 (*C*H_3_CH_2_), 26.9 (CH_2_), 28.5 [C(*C*H_3_)_3_], 28.9 (CH_2_), 29.0 (CH_2_), 29.1 (CH_2_), 29.3 (2CH_2_), 29.4 (2CH_2_), 29.5 (CH_2_), 33.7 (CH_2_=CH*C*H_2_), 38.1 (C-4), 47.5 (CH_2_N), 50.3 (C-5), 63.1 (C-1), 79.1 [*C*(CH_3_)_3_], 114.0 (*C*H_2_=CH), 139.1 (CH_2_=*C*H), 156.0 (N*C*O); HRMS (ESI) calcd for [C_23_H_45_NO_3_ + H^+^]: 384.3472, found: 384.3466.

*(S)-4-Ethyl-5-[(2-nitrobenzenesulfonyl)amino]-1-pentanol* (**12**). A solution of amino diol **7** (1.15 g, 4.58 mmol) in anhydrous MeOH (25 mL) containing 20% Pd(OH)_2_ (230 mg) was hydrogenated at 75 °C for 18 h under 10 bar of pressure. The catalyst was removed by filtration and washed with hot MeOH. The organic solution was concentrated, and the resulting crude amino alcohol **3** was dissolved in anhydrous CH_2_Cl_2_ (16 mL). 2-Nitrobenzenesulfonyl chloride (1.12 g, 5.0 mmol) and Et_3_N (0.7 mL, 5.0 mmol) were added, and the mixture was stirred at room temperature for 18 h. The organic solvent was removed under reduced pressure, and the residue was chromatographed (from 7:3 hexane-EtOAc to EtOAc) to give *N*-nosyl derivative **12** (1.1 g, 76% yield) as a colorless oil: [α]^22^_D_ +0.95 (*c* 0.84, MeOH); IR (ATR Pike) ν (cm^−1^): 3348 (OH, NH); ^1^H-NMR (400 MHz, CDCl_3_, COSY, *g*-HSQC): δ = 0.84 (t, *J* = 7.6 Hz, 3H, CH_3_), 1.30–1.40 (m, 4H, H-3, CH_3_C*H*_2_), 1.47–1.54 (m, 3H, H-2, H-4), 1.65 (br.s, 1H, OH), 3.02 (dt, *J* = 6.1, 3.7 Hz, 2H, H-5), 3.60 (t, *J* = 6.4 Hz, 2H, H-1), 5.41 (t, *J* = 6.0 Hz, 1H, NH), 7.76 (m, 2H, H-5Ns, H-6Ns), 7.85 (m, 1H, H-4Ns), 8.13 (m, 1H, H-3Ns); ^13^C-NMR (100.6 MHz, CDCl_3_): δ = 10.7 (CH_3_), 23.9 (CH_3_*C*H_2_), 26.9 (C-3), 29.3 (C-2), 33.1 (C-4), 46.2 (C-5), 62.8 (C-1), 125.3, 131.1 (C-3Ns, C-6Ns), 132.7 (C-4Ns), 133.5 (C-1Ns), 133.6 (C-5Ns), 148.0 (C-2Ns); HRMS (ESI) calcd for [C_13_H_20_N_2_O_5_S + H^+^]: 317.1166, found: 317.1161.

*(S)-4-Ethyl-5-[N-(2-nitrobenzenesulfonyl)-N-(10-undecenyl)amino]-1-pentanol* (**13**). 11-Bromo-1-undecene (0.80 mL, 3.65 mmol) was added to a suspension of *N*-nosyl derivative **12** (1.05 g, 3.3 mmol) and Cs_2_CO_3_ (1.3 g, 4.0 mmol) in anhydrous DMF (25 mL), and the resulting mixture was stirred at 55 °C for 3 h. The mixture was cooled to room temperature, poured into brine, and extracted with Et_2_O. The combined organic extracts were dried over anhydrous MgSO_4_, filtered, and concentrated under reduced pressure. Flash chromatography (7:3 hexane-EtOAc) of the residue gave alkene **13** (1.30 g, 84% yield) as a colorless oil: [α]^22^_D_ +1.82 (*c* 0.7, CH_2_Cl_2_); ^1^H-NMR (400 MHz, CDCl_3_, COSY, *g*-HSQC): δ = 0.85 (t, *J* = 7.6 Hz, 3H, CH_3_), 1.19–1.37 (m, 16H, 8CH_2_), 1.40–1.65 (m, 5H, H-4, CH_2_, CH_3_C*H*_2_), 2.02 (m, 2H, CH_2_=CHC*H*_2_), 3.19–3.25 (m, 4H, H-5, CH_2_N), 3.57 (t, *J* = 6.8 Hz, 2H, H-1), 4.95 (m, 2H, C*H*_2_=CH), 5.80 (m, 1H, CH_2_=C*H*), 7.62 (m, 1H, H-4Ns), 7.67 (m, 2H, C-5Ns, C-6Ns), 7.98 (m, 1H, H-3Ns); ^13^C-NMR (100.6 MHz, CDCl_3_): δ = 10.4 (CH_3_), 23.2 (CH_2_), 26.3 (CH_2_), 26.6 (CH_2_), 27.6 (CH_2_), 28.8 (CH_2_), 29.0 (CH_2_), 29.1 (CH_2_), 29.2 (CH_2_), 29.3 (CH_2_), 29.4 (CH_2_), 33.6 (CH_2_=CH*C*H_2_), 36.5 (C-4), 47.1 (CH_2_N), 50.7 (C-5), 62.8 (C-1), 114.0 (*C*H_2_=CH), 123.3 (C-3Ns), 130.6 (C-6Ns), 131.4 (C-4Ns), 133.3 (C-5Ns), 133.4 (C-1Ns), 139.0 (CH_2_=*C*H), 148.0 (C-2Ns); HRMS (ESI) calcd for [C_24_H_40_N_2_O_5_S + H^+^]: 469.2731, found: 469.2731.

*(S)-N-[2-Ethyl-5-hexenyl)-N-(2-nitrobenzenesulfonyl)-10-undecenamine* (**14**). Dess–Martin reagent (2.35 g, 5.54 mmol) was added to a solution of alcohol **13** (1.3 g, 2.77 mmol) in anhydrous CH_2_Cl_2_ (21 mL), and the mixture was stirred at room temperature for 1.5 h. Then, saturated aqueous Na_2_S_2_O_4_ (0.75 mL) and saturated aqueous NaHCO_3_ (0.75 mL) were added, and the resulting mixture was stirred for 1 h. The aqueous layer was extracted with CH_2_Cl_2_, and the combined organic extracts were washed with brine, dried over anhydrous Na_2_SO_4_, and filtered. The solvent was evaporated to give an aldehyde, which was used in the next step without further purification. *t*-BuOK (13.8 mL of a 1 M solution in THF, 13.8 mmol) was added to a solution of methyltriphenylphosphonium bromide (6.92 g, 19.4 mmol) in anhydrous THF (70 mL), and the solution was stirred at room temperature for 1 h. Then, a solution of the above aldehyde in anhydrous THF (10 mL) was added via cannula, and the resulting mixture was stirred at room temperature for 3 h. Saturated aqueous NH_4_Cl was added, and the mixture was extracted with EtOAc. The combined organic extracts were dried over anhydrous Na_2_SO_4_ and filtered, and the solvent was removed under reduced pressure. Flash chromatography (9:1 hexane-EtOAc) of the residue gave diene **14** (600 mg, 47% yield) as a colorless oil: [α]^22^_D_ +4.09 (*c* 2.1, MeOH); ^1^H-NMR (400 MHz, CDCl_3_, COSY, *g*-HSQC): δ = 0.84 (t, *J* = 7.5 Hz, 3H, CH_3_), 1.14–1.39 (m, 16H, 8CH_2_), 1.41–1.49 (m, 2H, CH_2_), 1.54–1.64 (m, 1H, CH), 1.95–2.10 (m, 4H, CH_2_=CHC*H*_2_), 3.14–3.26 (m,4H, 2CH_2_N), 4.91–5.02 (m, 4H, C*H*_2_=CHCH_2_), 5.74 (qt, *J* = 16.9, 10.0, 6.7, 6.7 Hz, 1H, CH_2_=C*H*), 5.80 (qt, *J* = 16.9, 10.1, 6.7, 6.7 Hz, 1H, CH_2_=C*H*), 7.59–7.62 (m, 1H, H-3Ns), 7.63–7.70 (m, 2H, H-5Ns, H-6Ns), 7.99–8.03 (m, 1H, H-4Ns). ^13^C-NMR (100.6 MHz, CDCl_3_): δ 10.3 (CH_3_), 23.2 (CH_2_), 26.6 (CH_2_), 27.5 (CH_2_), 28.9 (CH_2_), 29.0 (CH_2_), 29.1 (CH_2_), 29.3 (CH_2_), 29.4 (CH_2_), 29.5 (CH_2_), 30.6 (CH_2_=CH*C*H_2_), 33.8 (CH_2_=CH*C*H_2_), 36.0 (CH), 47.0 (CH_2_N), 50.7 (CH_2_N), 114.1 (*C*H_2_=CH), 114.7 (*C*H_2_=CH), 124.1 (C-3Ns), 130.9 (C-6Ns), 131.4 (C-4Ns), 133.2 (C-5Ns), 133.9 (C-1Ns), 138.5 (CH_2_=*C*H), 139.1 (CH_2_=*C*H), 148.0 (C-2Ns); HRMS (ESI) calcd for [C_25_H_40_N_2_O_4_S + H^+^]: 465.2782, found 465.2776.

*(S)-3-Ethyl-1-(2-nitrobenzenesulfonyl)azacyclohexadec-6-ene* (**15**). A solution of diene **14** (70 mg, 0.15 mmol) in anhydrous CH_2_Cl_2_ (15 mL) was added to a solution of second-generation Grubbs catalyst (19 mg, 0.022 mmol) in CH_2_Cl_2_ (750 mL). The mixture was stirred at reflux temperature for 14 h. The solvent was removed under reduced pressure, and the resulting residue was chromatographed (95:5 hexane-EtOAc) to yield macrocycle **15** (46 mg, 70% yield) as a 88:12 mixture (calculated by GC/MS) of *E/Z* diastereoisomers. Spectroscopic data of *E* diastereoisomer (from a mixture): ^1^H-NMR (400 MHz, CDCl_3_, COSY, *g*-HSQC): δ = 0.83 (t, *J* = 7.5 Hz, 3H, CH_3_), 1.00–1.11 (m, 1H, H-4), 1.18–1.44 (m, 17H, H-4, 8CH_2_), 1.46–1.51 (m, 2H, CH_2_), 1.67–1.80 (m, 1H, H-3), 1.97–2.19 (m, 4H, H-5, H-8), 3.07 (dd, *J* = 13.8, 7.5 Hz, H-2), 3.11–3.15 (m, 1H, H-16), 3.21–3.26 (m, 1H, H-16), 3.28 (dd, *J* = 13.8, 7.5 Hz, H-2), 5.25–5.41 (m, 2H, H-6, H-7), 7.57–7.62 (m, 1H, H-3Ns), 7.63–7.73 (m, 2H, H-5Ns, H-6Ns), 7.93–8.04 (m, 1H, H-4Ns); ^13^C-NMR (100.6 MHz, CDCl_3_): δ = 9.6 (CH_3_), 21.9 (CH_2_), 24.3 (CH_2_), 25.7 (CH_2_), 26.1 (CH_2_), 26.1 (CH_2_), 26.7 (CH_2_), 26.7 (CH_2_), 27.3 (CH_2_), 28.8 (CH_2_=CH*C*H_2_), 29.6 (CH_2_), 30.9 (CH_2_=CH*C*H_2_), 33.3 (C-3), 46.7 (C-16), 51.3 (C-2), 124.0 (C-3Ns), 130.2 (CH=), 130.7 (C-6Ns), 131.3 (C-4Ns), 131.4 (C-5Ns), 133.2 (CH=), 133.4 (C-1Ns), 148.1 (C-2Ns); HRMS (ESI) calcd for [C_23_H_36_N_2_O_4_S + H^+^]: 437.2469, found 437.2459.

*(S)-3-Ethylazacyclohexadec-6-ene* (**16**). Thiophenol (0.024 mL, 0.34 mmol) and K_2_CO_3_ (82 mg, 0.59 mmol) were added to a solution of cycloalkene **15** (86 mg, 0.20 mmol) in anhydrous DMF (4 mL), and the mixture was stirred at room temperature for 14 h. Then, 2 M aqueous NaOH was added, and the mixture was extracted with CH_2_Cl_2_. The combined organic extracts were dried over anhydrous MgSO_4_, filtered, and concentrated under reduced pressure. Flash chromatography (from 95:5 hexane-EtOAc to 8:2 EtOAc-Et_3_N) of the residue gave macrocycle **16** (23 mg, 45% yield) as a mixture of *E/Z* diastereoisomers as a brown oil. Spectroscopic data of *E* diastereoisomer (from a mixture): ^1^H-NMR (400 MHz, CDCl_3_, COSY, *g*-HSQC): δ = 0.87 (t, *J* = 7.5 Hz, 3H, CH_3_), 1.25–1.44 (m, 16H, 8CH_2_), 1.47–1.56 (m, 3H, CH_2_), 1.94–2.12 (m, 4H, H-5, H-8), 2.41 (dd, *J* = 12.1, 7.5 Hz, 1H, H-2), 2.51–2.57 (dd, *J* = 12.0, 6.4 Hz, 1H, H-16), 2.52–2.58 (dd, *J* = 12.1, 5.9 Hz, 1H, H-2), 2.62–2.68 (dd, *J* = 12.0, 5.7 Hz, 1H, H-16), 5.36–5.39 (m, 2H, H-6, H-7); ^13^C-NMR (100.6 MHz, CDCl_3_): δ = 10.8 (CH_3_), 24.4 (CH_2_), 25.2 (CH_2_), 26.2 (CH_2_), 26.3 (CH_2_), 27.2 (CH_2_), 27.3 (CH_2_), 28.0 (CH_2_), 28.2 (CH_2_), 29.1 (CH_2_=CH*C*H_2_), 30.8 (CH_2_), 31.8 (CH_2_=CH*C*H_2_), 36.9 (C-3), 47.6 (C-16), 52.3 (C-2), 130.8 (CH=), 130.9 (CH=); HRMS (ESI) calcd for [C_17_H_33_N + H^+^]: 252.2686, found 252.2689.

*(S)-3-Ethylazacyclohexadecane* (**5**). A solution of cycloalkene **16** (19 mg, 0.076 mmol) in anhydrous MeOH (10 mL) containing 25% Pd/C (5 mg) was hydrogenated at room temperature for 14 h under 10 bar of pressure. The catalyst was removed by filtration and washed with hot MeOH, and the organic solution was concentrated under reduced pressure. Flash chromatography (from 95:5 hexane-EtOAc to 8:2 EtOAc-Et_3_N) of the residue gave **5** (10 mg, 52% yield) as a brown oil: [α]^22^_D_ −6.75 (*c* 0.15, MeOH); ^1^H-NMR (300 MHz, CDCl_3_): δ = 0.90 (t, *J* = 7.5 Hz, 3H, CH*_3_*), 1.25–1.41 (m, 24H, 12CH_2_), 1.50–1.70 (m, 3H, H-3, CH_2_), 2.40 (m, 2H, H-2), 2.60–2.85 (m, 2H, H-16); ^13^C-NMR (100.6 MHz, CDCl_3_): δ = 10.8 (CH_3_), 24.8 (CH_2_), 24.9 (CH_2_), 25.0 (CH_2_), 26.0 (CH_2_), 26.1 (CH_2_), 26.2 (CH_2_), 26.3 (CH_2_), 26.4 (CH_2_), 26.5 (CH_2_), 27.1 (CH_2_), 27.4 (CH_2_), 29.7 (CH_2_), 29.8 (CH_2_), 35.6 (C-3), 46.1 (C-16), 49.1 (C-2); HRMS (ESI) calcd for [C_17_H_35_N + H^+^]: 254.2842, found 254.2842.

## Supplementary Materials

The following are available online, Copies of ^1^H and ^13^C NMR spectra.
