# Supplementary material for: Enantioselective Synthesis of the Ethyl Analog of the Marine Alkaloid Haliclorensin C
Source: Molecules. 2019 Mar 18;24(6):1069. doi: 10.3390/molecules24061069 (PMC6470606; doi:10.3390/molecules24061069)

# Enantioselective Synthesis of the Ethyl Analog of the Marine Alkaloid Haliclorensin C

**Guillaume Guignard, Núria Llor, David Pubill, Joan Bosch and Mercedes Amat**

Supporting Information Available

Copies of $^{1}H$ and $^{13}C$ NMR spectra pages: S2-S12

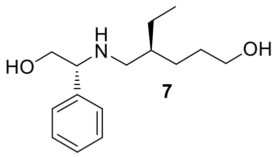


**7**

$^{1}$H NMR (400 MHz, $CDCl_3$)

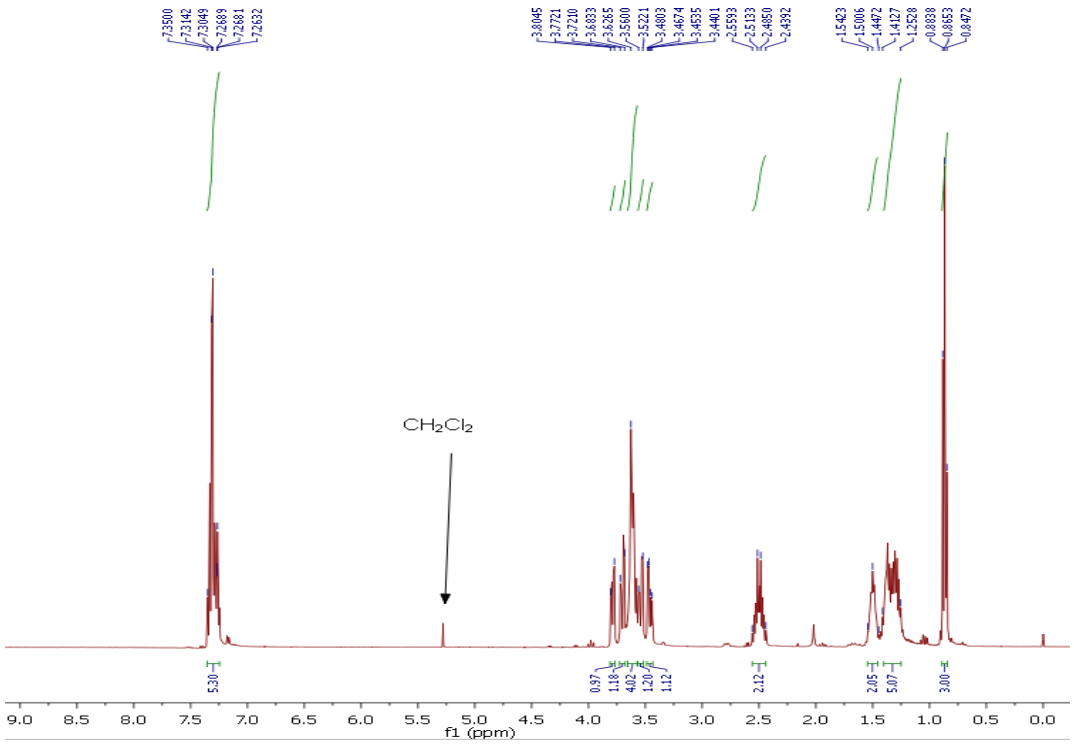


$^{13}$C NMR (100.6 MHz, $CDCl_3$)

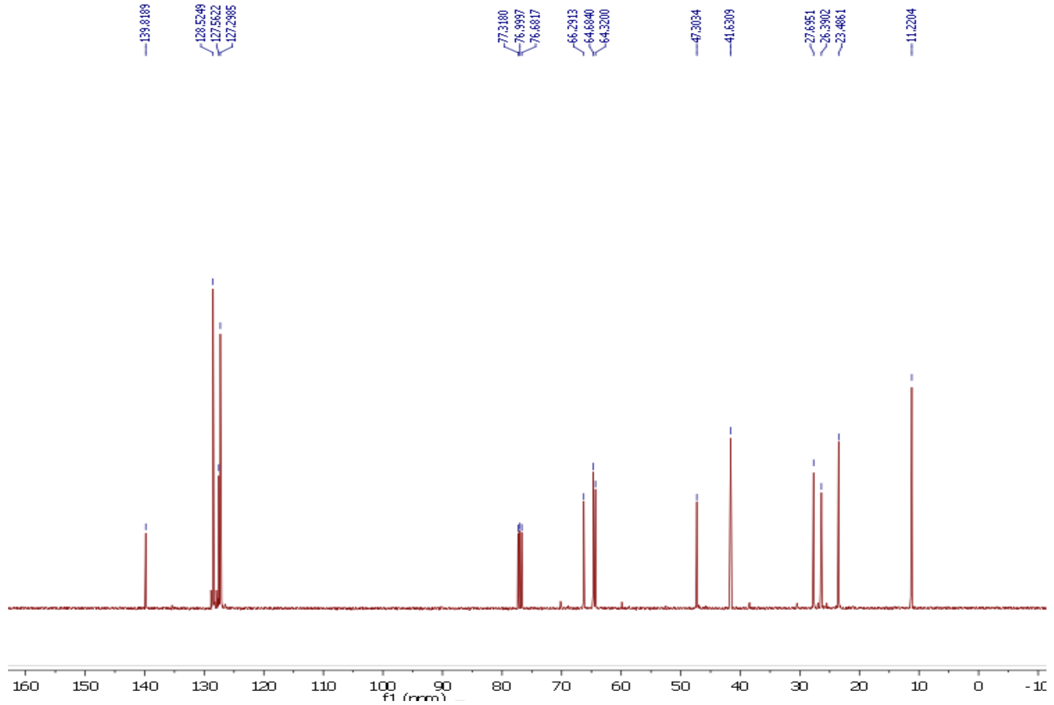

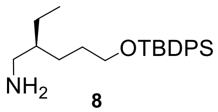


**8**

$^1$H NMR (400 MHz, $CDCl_3$)

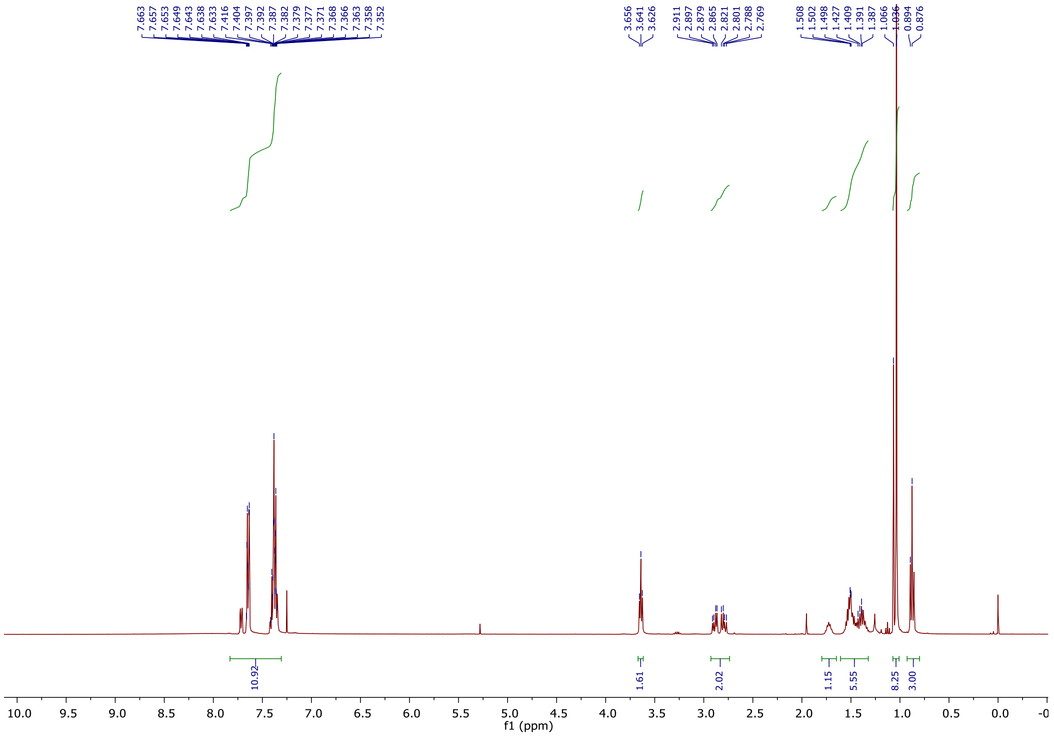


$^{13}$C NMR (100.6 MHz, $CDCl_3$)

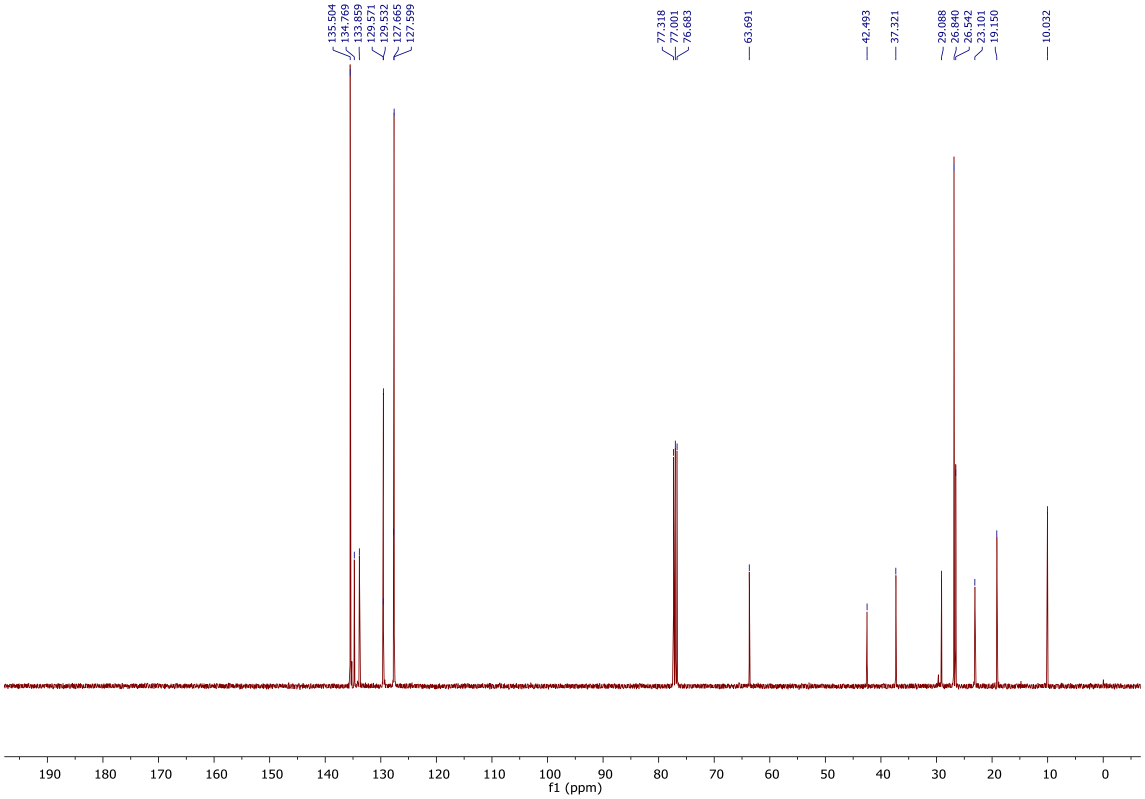

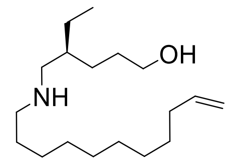


**9**

$^{1}$H NMR (400 MHz, $CDCl_3$)

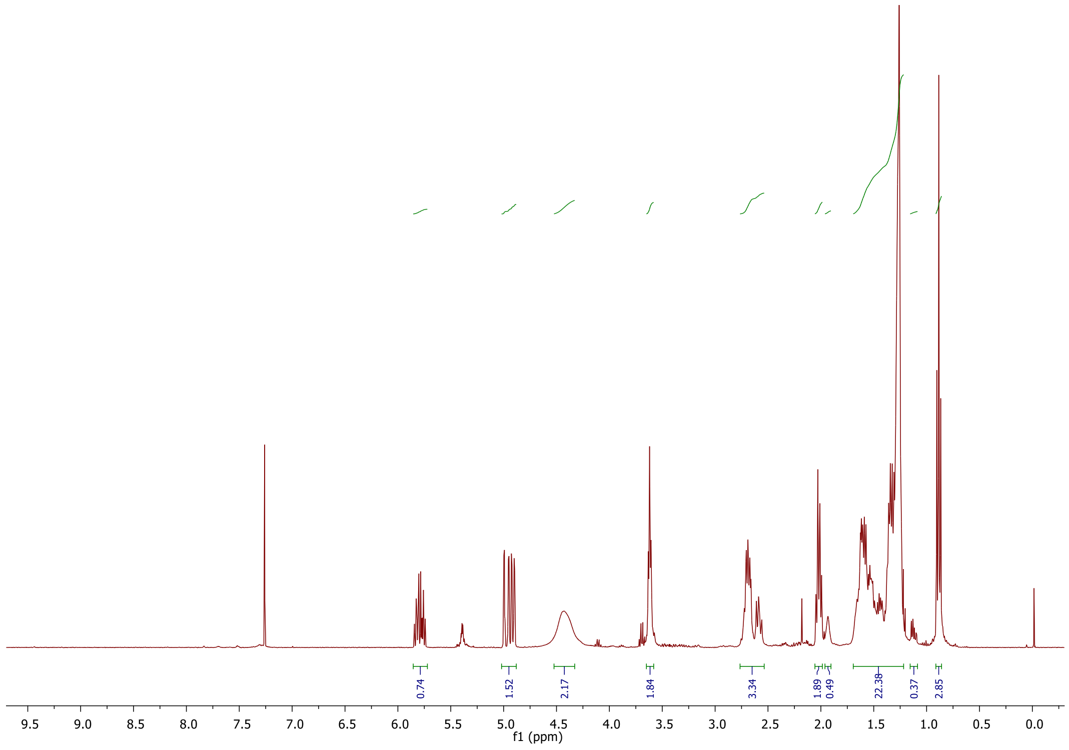


$^{13}$C NMR (100.6 MHz, $CDCl_3$)

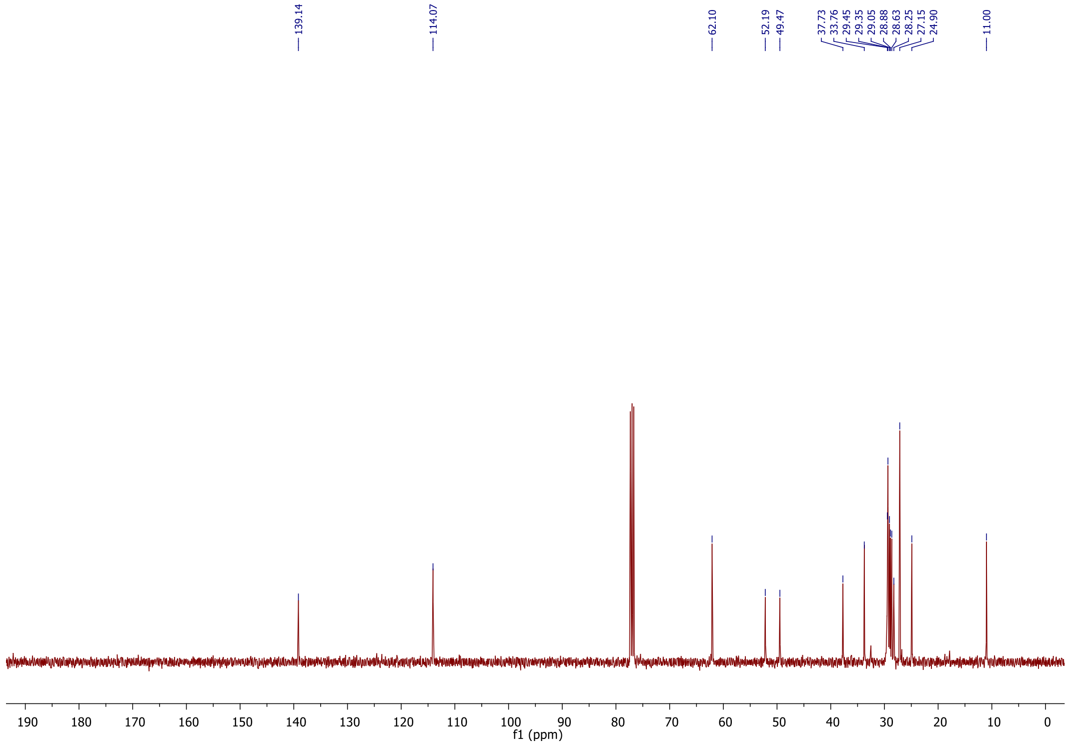

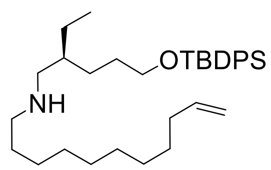


**10**

<sup></sup>

$^{1}$H NMR (400 MHz, $CDCl_3$)

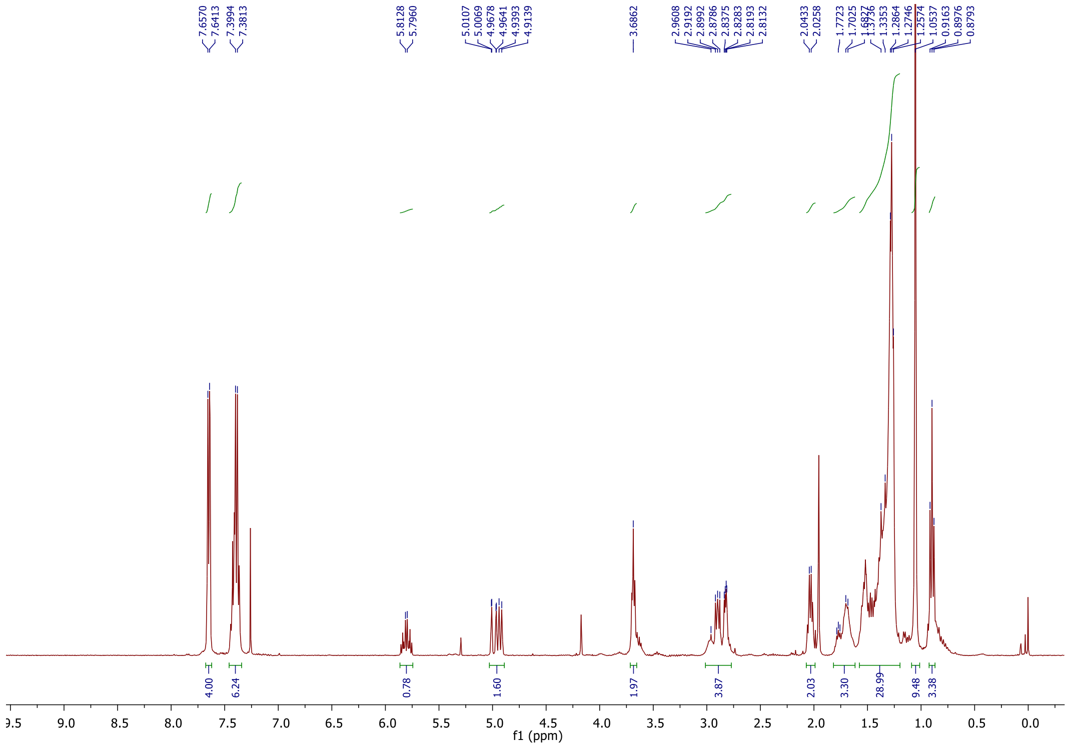


$^{13}$C NMR (100.6 MHz, $CDCl_3$)

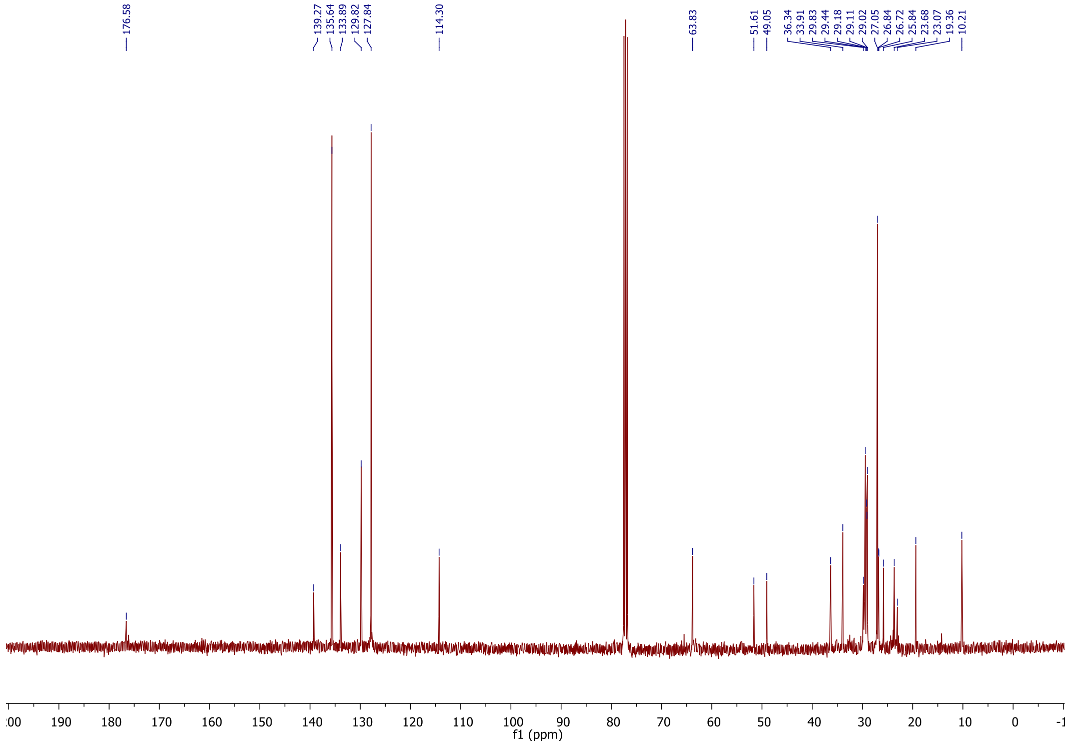

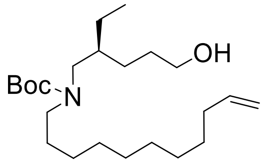


**11**

$^{1}$H NMR (400 MHz, $CDCl_3$)

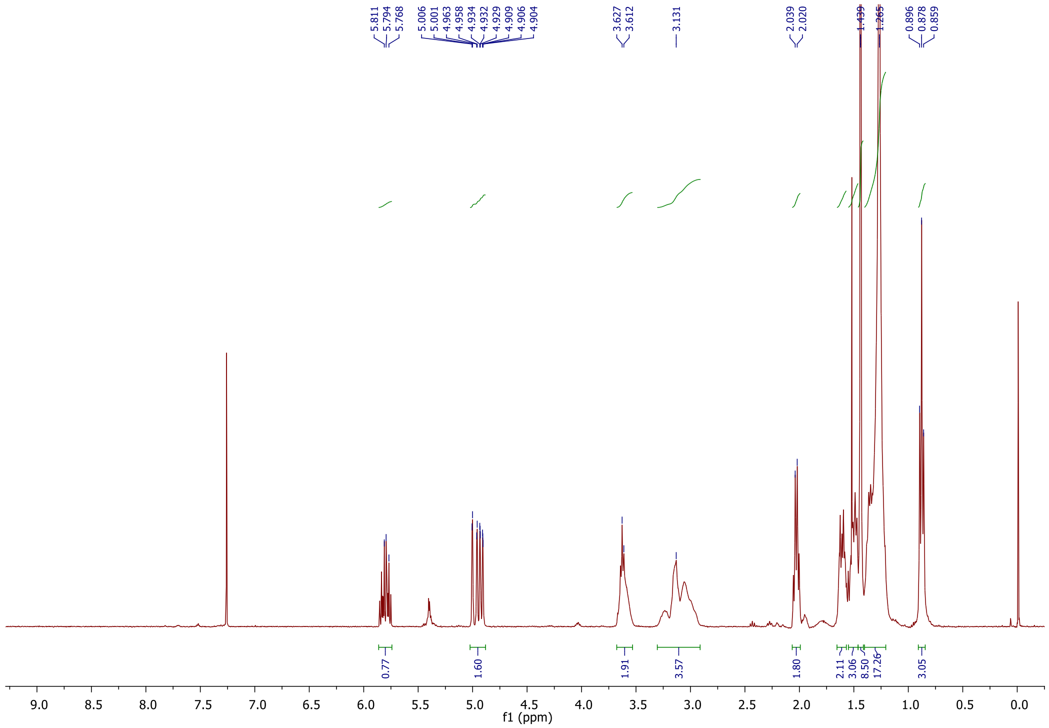


$^{13}$C NMR (100.6 MHz, $CDCl_3$)

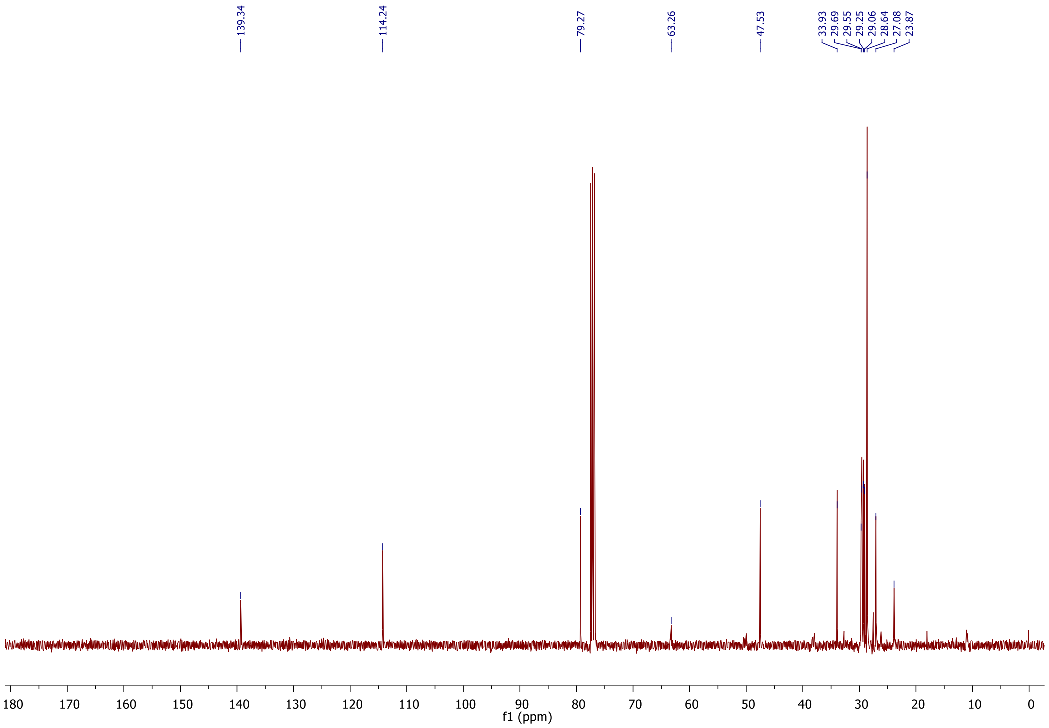

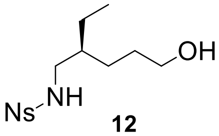


**12**

$^{1}$H NMR (400 MHz, $CDCl_3$)

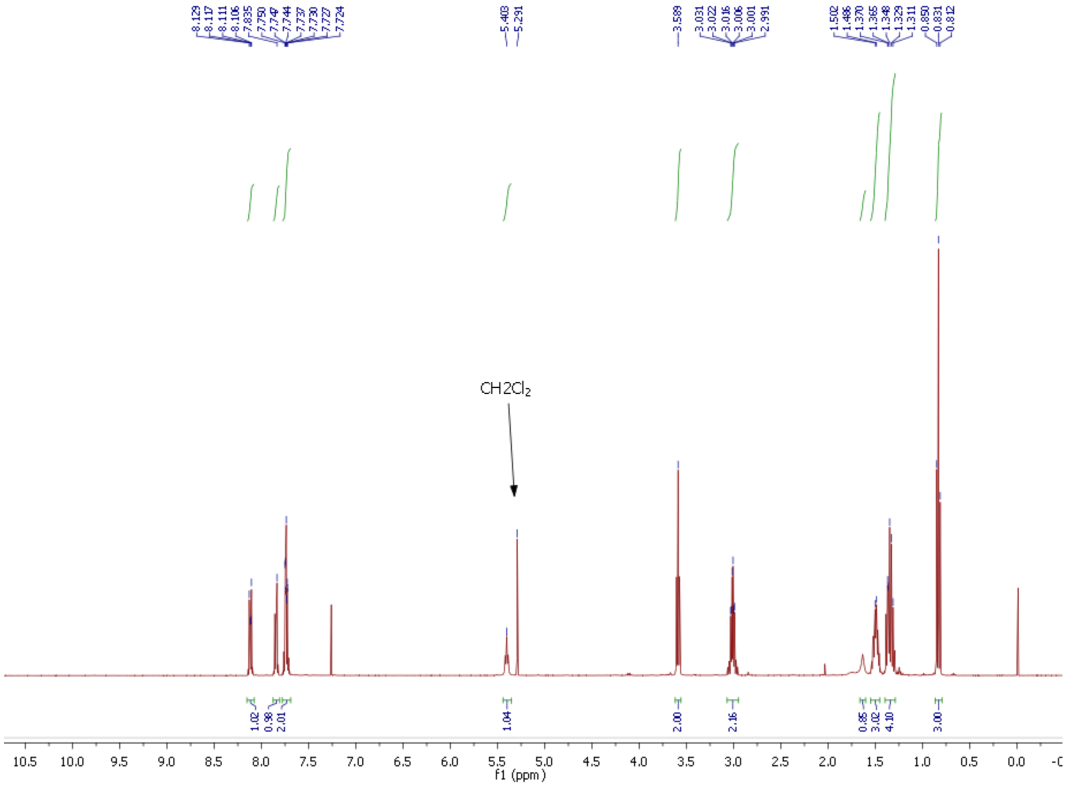


$^{13}$C NMR (100.6 MHz, $CDCl_3$)

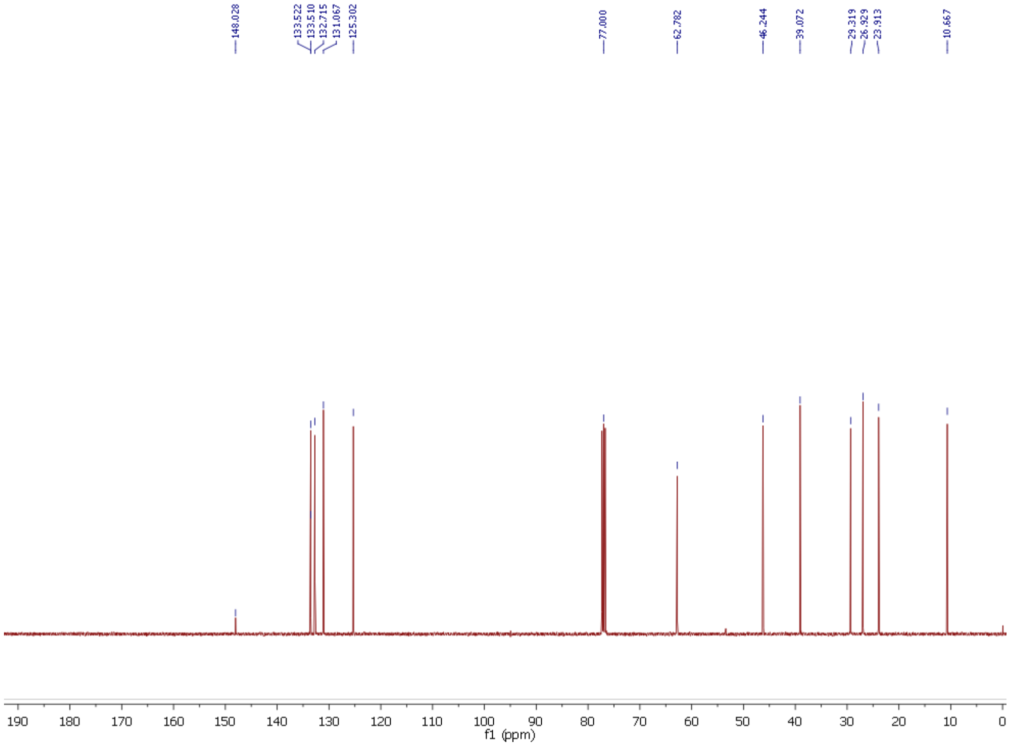

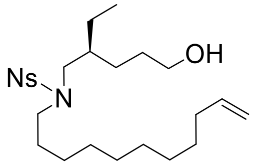


**13**

$^{1}H$ NMR (400 MHz, $CDCl_3$)

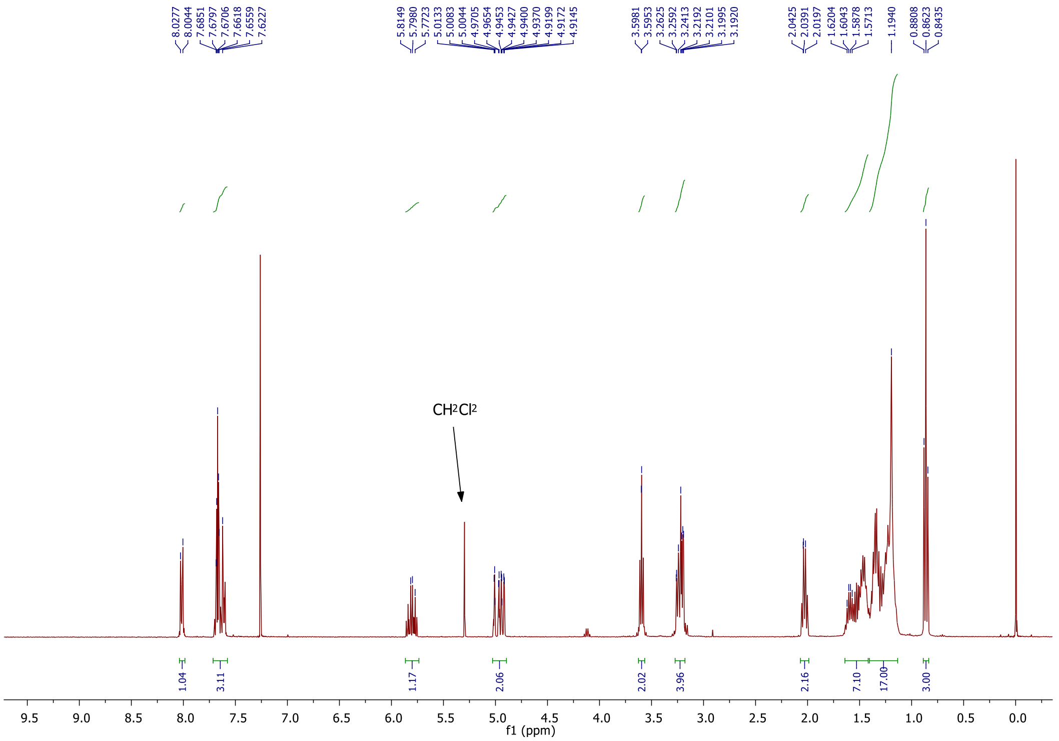


$^{13}C$ NMR (100.6 MHz, $CDCl_3$)

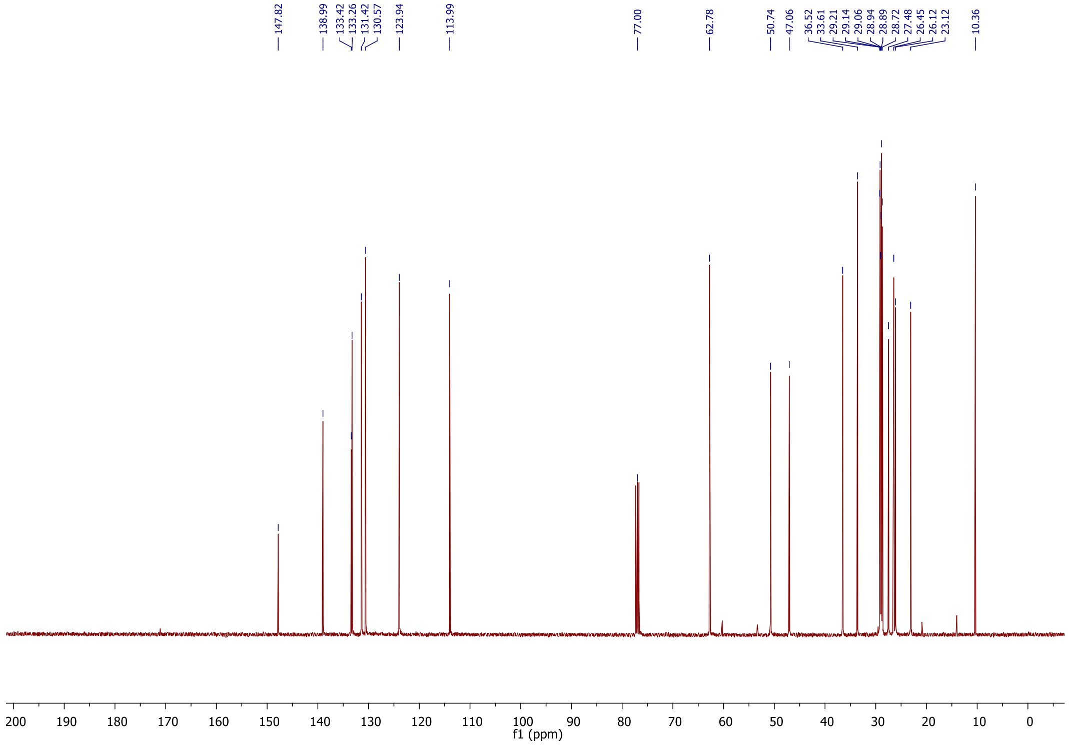

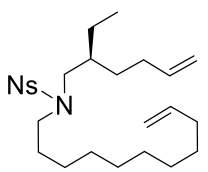


**14**

$^{1}$H NMR (400 MHz, $CDCl_3$)

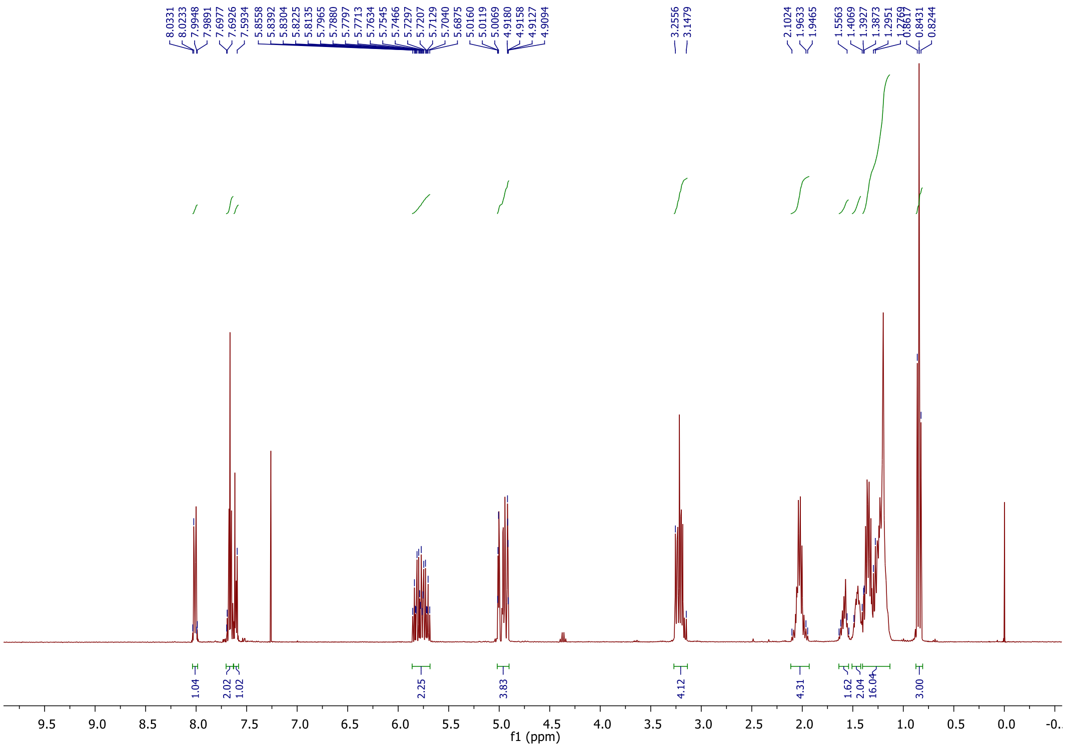


$^{13}$C NMR (100.6 MHz, $CDCl_3$)

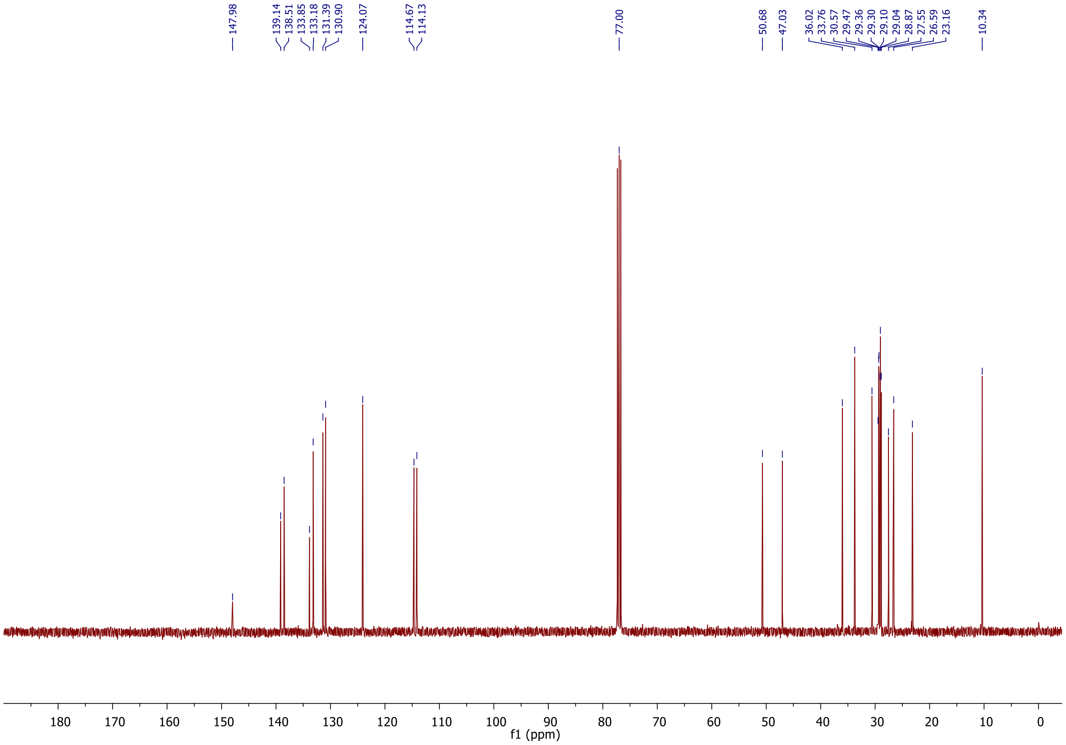

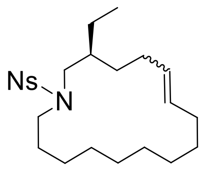


**15**

$^{1}$H NMR (400 MHz, $CDCl_3$) mixture of *E/Z* diastereoisomers 88:12 (GC/MS)

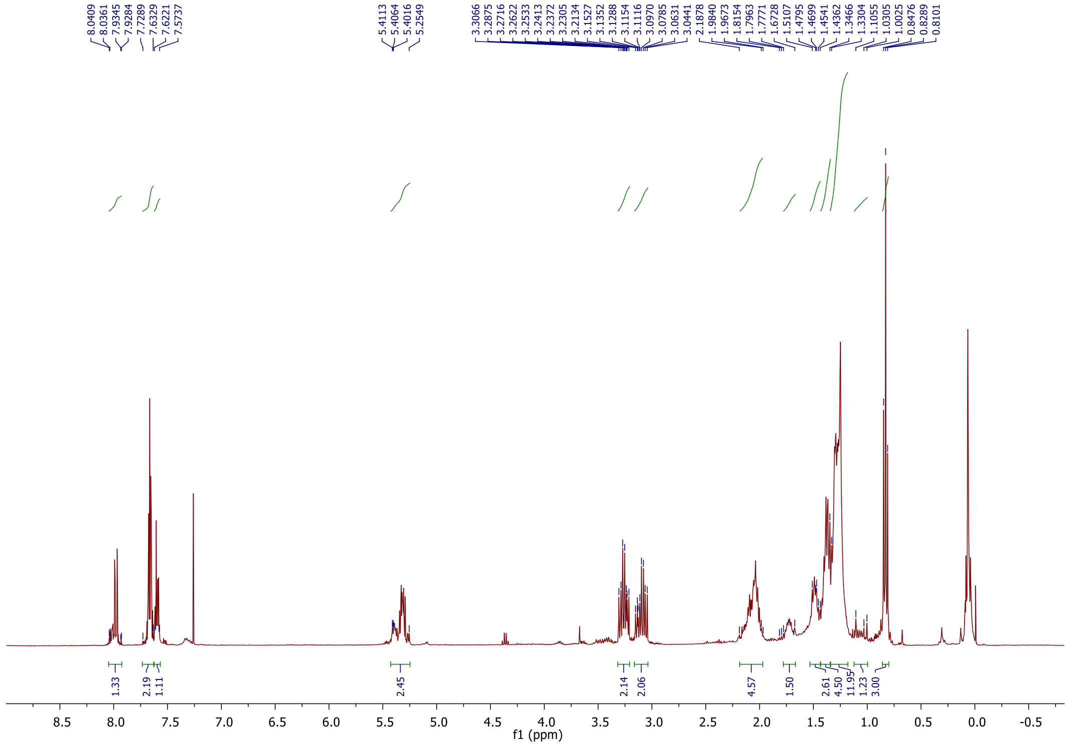


$^{13}$C NMR (100.6 MHz, $CDCl_3$)

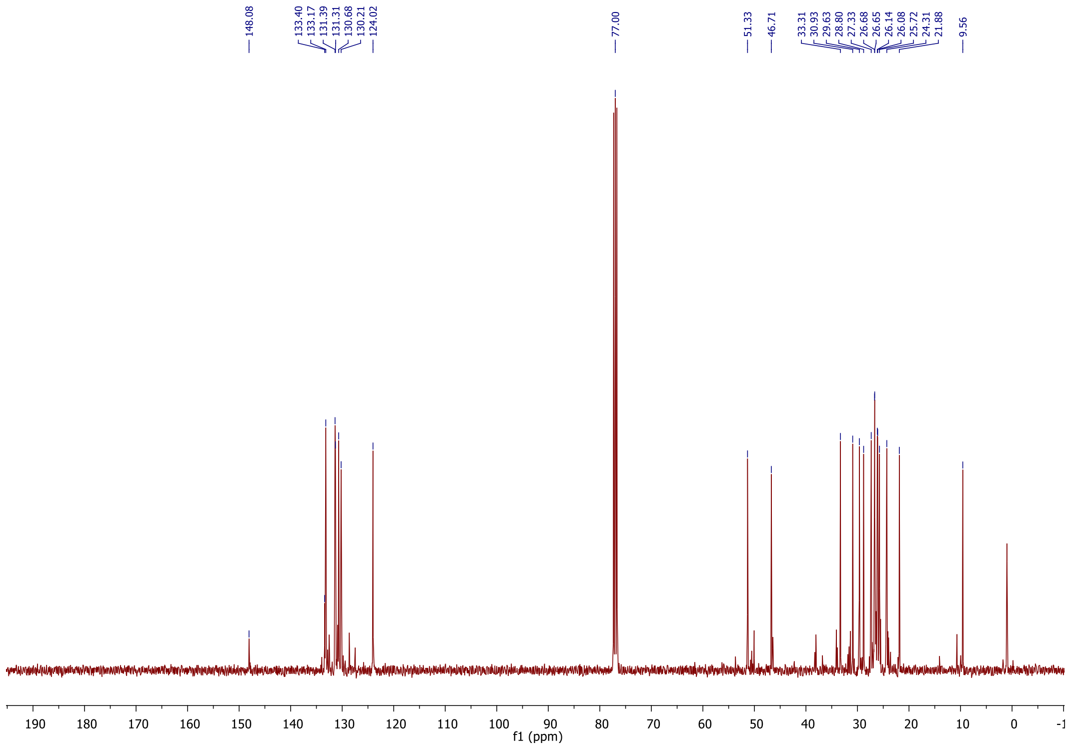

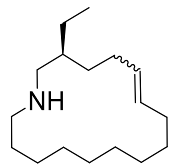


**16**

$^{1}$H NMR (400 MHz, $CDCl_3$) mixture of *E/Z* diastereoisomers

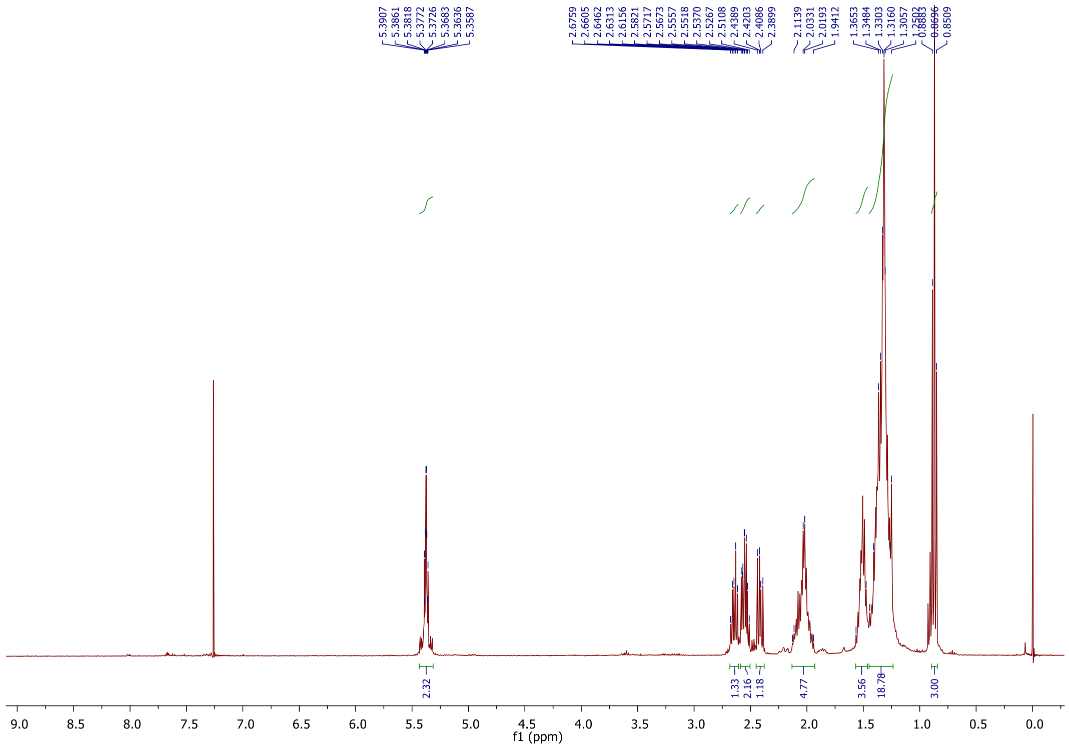


$^{13}$C NMR (100.6 MHz, $CDCl_3$)

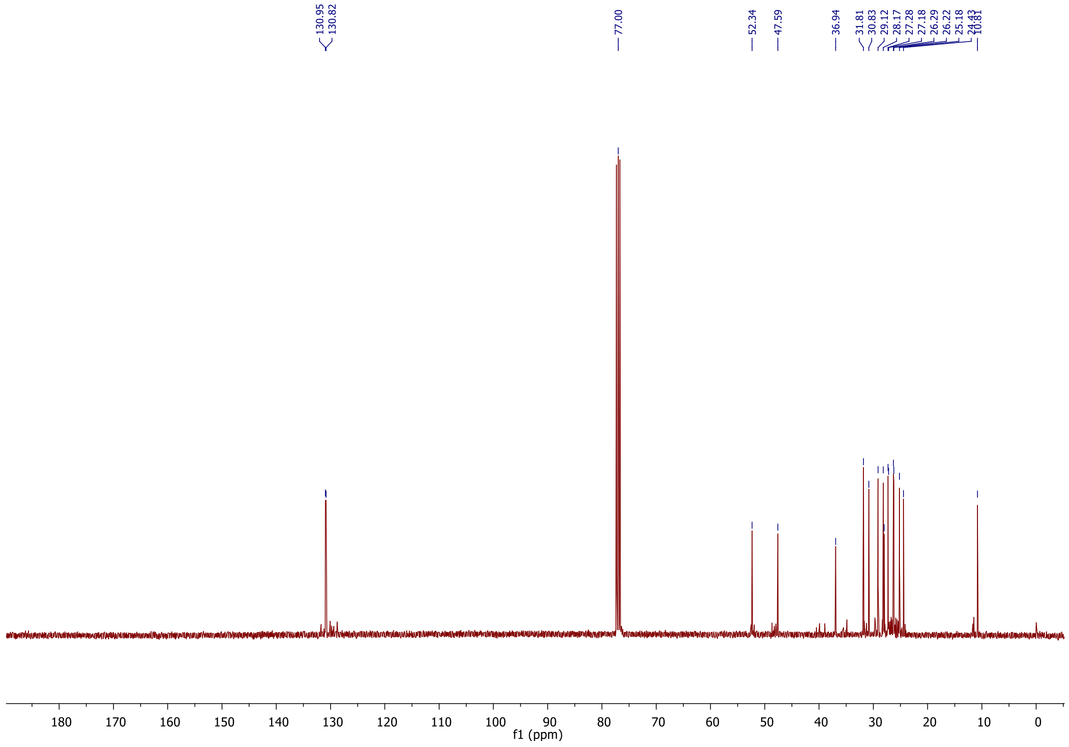

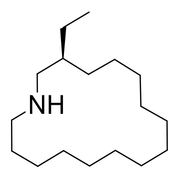


**5**

$^{1}$H NMR (400 MHz, $CDCl_3$)

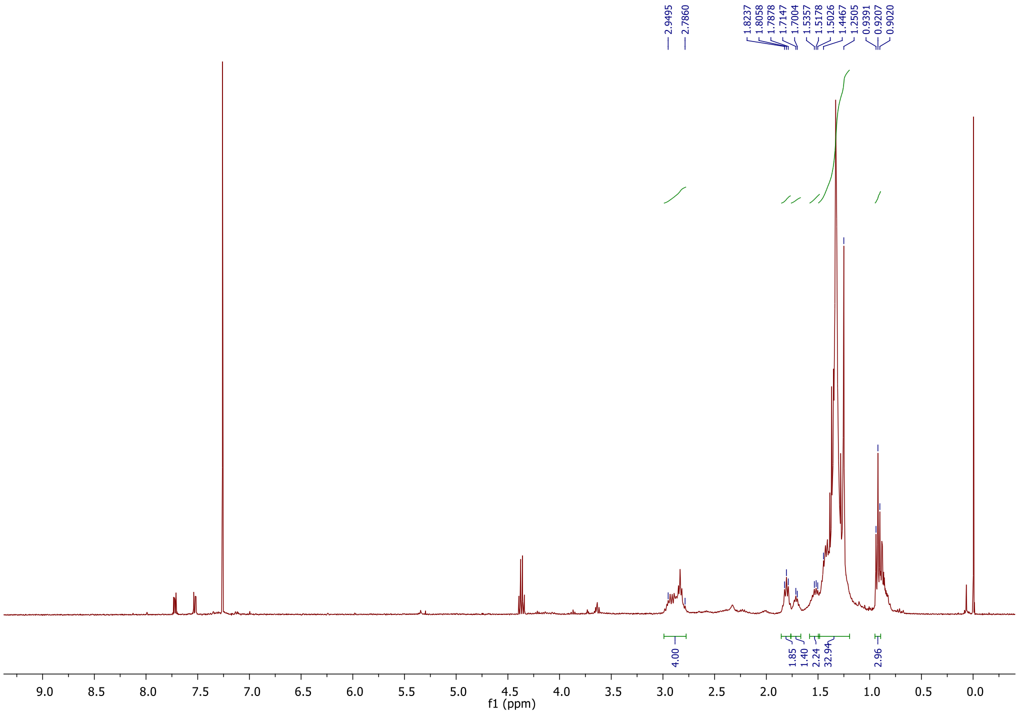


$^{13}$C NMR (100.6 MHz, $CDCl_3$)

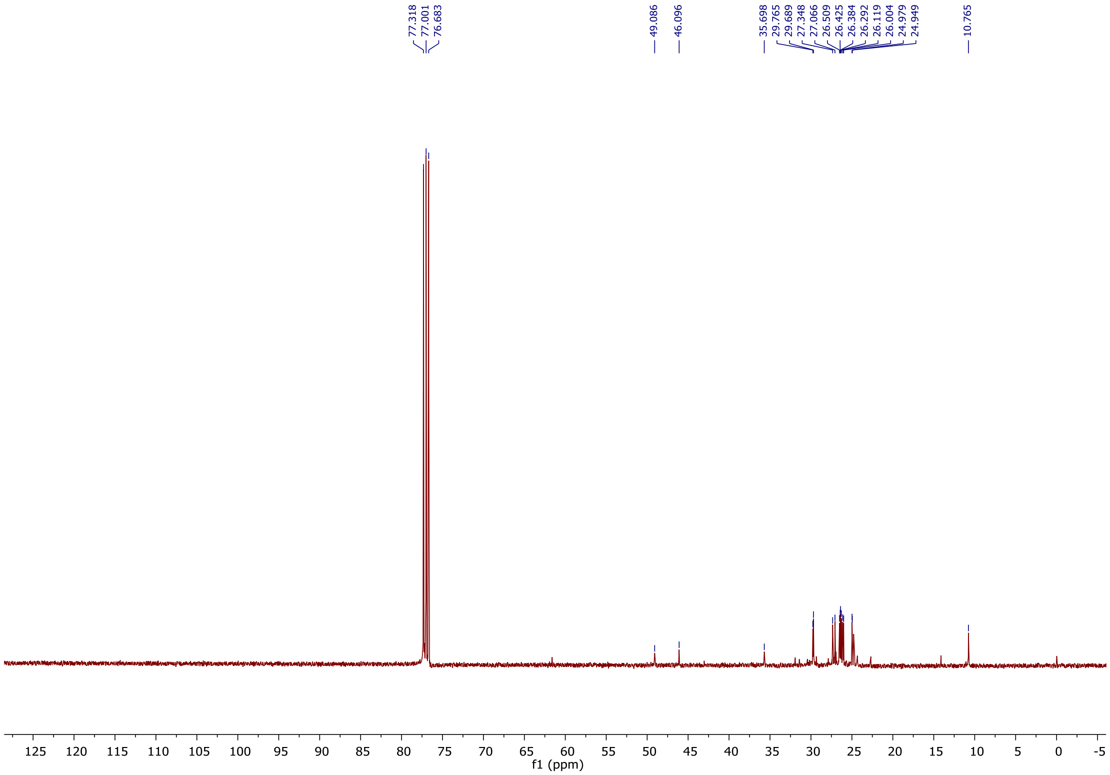

Supplement: Supplementary file 1 [file molecules-24-01069-s001.pdf]
